# Nobiletin Ameliorates Cellular Damage and Stress Response and Restores Neuronal Identity Altered by Sodium Arsenate Exposure in Human iPSCs-Derived hNPCs

**DOI:** 10.3390/ph15050593

**Published:** 2022-05-12

**Authors:** Sadaf Jahan, Uzair Ahmad Ansari, Arif Jamal Siddiqui, Danish Iqbal, Johra Khan, Saeed Banawas, Bader Alshehri, Mohammed Merae Alshahrani, Suliman A. Alsagaby, Neeru Singh Redhu, Aditya Bhushan Pant

**Affiliations:** 1 Department of Medical Laboratory Sciences, College of Applied Medical Sciences, Majmaah University, Al-Majmaah 11952, Saudi Arabia; da.mohammed@mu.edu.sa (D.I.); j.khan@mu.edu.sa (J.K.); s.banawas@mu.edu.sa (S.B.); b.alshehri@mu.edu.sa (B.A.); s.alsaqaby@mu.edu.sa (S.A.A.); 2 Developmental Toxicology Laboratory, Systems Toxicology & Health Risk Assessment Group, CSIR-Indian Institute of Toxicology Research (CSIR-IITR), Vishvigyan Bhavan, 31, Mahatma Gandhi Marg, P.O. Box No. 80, Lucknow 226001, Uttar Pradesh, India; ansari.uzair009@gmail.com (U.A.A.); ab.abpant@gmail.com (A.B.P.); 3 Academy of Scientific and Innovative Research (AcSIR), Ghaziabad 201002, Uttar Pradesh, India; 4 Department of Biology, College of Science, University of Hail, P.O. Box 2440, Hail 55476, Saudi Arabia; arifjamal13@gmail.com; 5 Department of Biomedical Sciences, Oregon State University, Corvallis, OR 97331, USA; 6 Department of Clinical Laboratory Sciences, Faculty of Applied Medical Sciences, Najran University, 1988, Najran 61441, Saudi Arabia; mmalshahrani@nu.edu.sa; 7 Department of Molecular Biology, Biotechnology and Bioinformatics, Chaudhary Charan Singh Haryana Agricultural University, Hisar 125004, Haryana, India; neru.redhu95@gmail.com

**Keywords:** neurological complications, nobiletin, sodium arsenate, ROS, stress granule, neuronal markers

## Abstract

Environmental exposure to arsenic has been profoundly associated with chronic systemic disorders, such as neurodegeneration, in both experimental models and clinical studies. The neuronal cells of the brain and the nervous system have a limited regeneration capacity, thus making them more vulnerable to exposure to xenobiotics, leading to long-lasting disabilities. The functional and anatomical complexity of these cells hinders the complete understanding of the mechanisms of neurodegeneration and neuroprotection. The present investigations aimed to evaluate the neuroprotective efficacy of a herbal formulation of Nobiletin (NOB) against the toxic insult induced by sodium arsenate (NA) in human neural progenitor cells (hNPCs) derived from human induced pluripotent stem cells (hiPSCs). Prior to the neuroprotective experiments, biologically safe doses of both NOB and NA were ascertained using standard endpoints of cytotoxicity. Thereafter, the hNPCs were exposed to either NOB (50 μM) or NA (50 μM) and co-exposed to biologically safe concentrations of NA (50 μM) with NOB (50 μM) for a period of up to 48 h. NOB treatment restored the morphological damage (neurite damage), the levels of stress granule G3BP1 (Ras-GTPase-activating protein (SH3 domain)-binding protein) and TIA1 (T cell-restricted intracellular antigen), and the expression of neuronal markers (Tuj1, Nestin, MAP2, and PAX6) when compared to NA-exposed cells. A substantial restoration of reactive oxygen species and mitochondrial membrane potential was also witnessed in the co-exposure group (NA + NOB) in comparison to the NA-exposed group. The findings suggest that NOB possesses a significant restorative/protective potential against the NA challenge in hNPCs under experimental conditions and imply that nobiletin may impart a potential therapeutic impact if studied adequately using in vivo studies.

## 1. Introduction

Arsenic (As) is a ubiquitously dispersed environmental toxicant that has become a public health issue owing to its potential to harm human health. The threat of occupational and natural exposure to As has greatly increased because of its widespread industrial use for manufacturing alloys, ceramics, insecticides, and paints [1,2,3]. According to a WHO report, As contamination of groundwater affects roughly 140 million people in 50 countries all across the globe [4]. Chronic exposure to As through drinking water results in both cancerous and non-cancerous endpoint diseases. The most prevalent cancers in afflicted people are skin, lung, and urinary bladder cancers [5]. However, several non-cancer diseases are also emerging as a threat to human health, e.g., diabetes, hypertension, cardiovascular disease, and neurological disorders [6]. In a cohort study on the chronically As-exposed population in Bangladesh, the authors reported cognitive impairment and neurodevelopmental abnormalities in children affected by perinatal exposure to As [7]. It has been proven that As penetrates the blood–brain barrier and induces oxidative stress, which causes a variety of neurological problems in children, especially at lower doses [8,9].

As-induced oxidative stress can cause mitochondrial malfunction and neuronal death. Exposure to As leads to the generation of reactive oxygen species (ROS) and disrupts the antioxidant defense mechanisms [10]. The most widely acknowledged mechanism in As-induced neurotoxicity is increased oxidative stress accompanied by diminished antioxidant capacity. Demyelination and physiological changes in the axons of peripheral nerves were reported to be related to enhanced oxidative damage in an animal model of As exposure [11]. Furthermore, As administration promotes oxidative stress in both in vivo as well as in vitro glial and neuronal cell types [12,13,14]. The central nervous system and the brain are prone to oxidative damage because of their high levels of oxygen expenditure for higher energy needs, as well as high quantities of PUFAs (polyunsaturated fatty acids) and lower levels of antioxidant defense components [15,16]. As-induced stress also results in the generation of stress granules (SGs) [17]. The assembly of SGs with G3BP1 (Ras-GTPase-activating protein (SH3 domain)-binding protein) and TIA1 (T cell-restricted intracellular antigen) has been well characterized in response to different metabolic and oxidative stressors in neuronal systems. To survive a stressful situation, cells must conserve energy by silencing or limiting protein production. The development of SGs is one method for regulating protein synthesis. These granules comprise scaffold and RNA-binding proteins that protect the cell from toxicant-induced damage while keeping mRNAs translationally silent [18,19,20]. The stress granule response has also been studied with respect to As-mediated toxicity in brain cells. Therefore, the accumulation and clearance of stress granules may provide a quantifiable measure of As-induced toxicity and its potential alleviation by way of naturally occurring antioxidant compounds or plant extracts.

Antioxidant agents have been proven to safeguard brain cells from As-induced oxidative impairment. The protective effects of pharmacological drugs and herbal extracts in As-induced neurotoxicity have been widely addressed [21]. Nobiletin (NOB) is one such example; it is a primary polymethoxyflavone found in the peels of citrus fruit such as limes, oranges, lemons, and mandarins [22]. Antioxidant, anti-inflammatory, anti-diabetic, and anti-carcinogenic actions have all been ascribed to nobiletin [23,24]. Nobiletin’s neuroprotective properties have been proven in a number of recent studies. In numerous animal models for AD, such as APP-SL 7–5 Tg mice, olfactory bulbectomy mice, and 3XTg-AD mice, nobiletin decreased Aβ-stimulated cognitive deterioration. The amelioration of the Akt/cAMP-response element-binding protein (CREP)/Bcl-2 pathway in damaged brains of ischemic Sprague-Dawley rats was observed under the therapeutic influence of NOB [25,26,27]. A notable restoration in the decreased levels of the glutathione (GSH)/glutathione disulfide (GSSG) ratio (an oxidative stress indicator) was observed during NOB treatment in the SAMP8 AD mouse model. The functioning of intracellular antioxidant enzymes such as manganese-superoxide dismutase and glutathione peroxidase were boosted by the action of nobiletin [28]. It was observed in a rat middle cerebral artery occlusion model that NOB dramatically boosted the levels of SOD1, Nrf2, GSH, and HO-1; in addition, the intensity of NF-κB, MDA, and MMP9 decreased [24]. In vitro studies on HT22 cells, PC12 cells, and SK-N-SH cells investigated the impact of nobiletin on oxidative stress and endoplasmic reticulum stress. Nobiletin showed neuroprotective effects on PC12 cells against hydrogen peroxide (H_2_O_2_)-induced apoptosis and restored the H_2_O_2_-induced decline in GSH and SOD levels [29,30]. Masjosthusmann et al., in 2019, investigated the detrimental toxicity of sodium arsenite (NaAsO_2_) on progenitor cells of rat and human brains (rNPCs and hNPCs, respectively). According to the authors, NaAsO_2_ impaired neurogenesis, exodus, and oligodendrogenesis in differentiating hNPCs and rNPCs [31]. Another study reported that in arsenic-exposed rats, the expression of numerous key genes required for normal maturation and axonal development, such as doublecortin, tenascin R, Mtap2, and Robo1, was found to be significantly downregulated. This adds to the theory that arsenic affects the capacity of hNPCs to grow into mature neurons, perhaps contributing to the reduction in the number of differentiating neurons [32]. Since arsenic toxicity is well reported in early stages of neurodevelopment, we employed hNPCs to assess arsenic-induced neurotoxicity [33].

To ameliorate neuronal damage, researchers are focusing on herbal compounds that have proven promising against neurotoxicity because of their anti-oxidative and neuroprotective efficacy and minimal adverse effects. The available quantum of literature highlights the multifaceted physiological activity of NOB and its neuroprotective effects on the brain. Here, we examined the impact of NOB against NA-induced neurotoxicity in hNPCs. We examined sodium arsenate-induced oxidative stress and neuronal damage in terms of the accumulation of the stress granule markers G3BP1 and TIA1 and also in terms of changes in the expression of neuronal markers. To the best of our knowledge, we report here, for the first time, NA-induced neurotoxicity on hNPCs and the neuroprotective effects of NOB on NA-challenged hNPCs.

## 2. Results

### 2.1. Cell Viability

**Figure 1:**
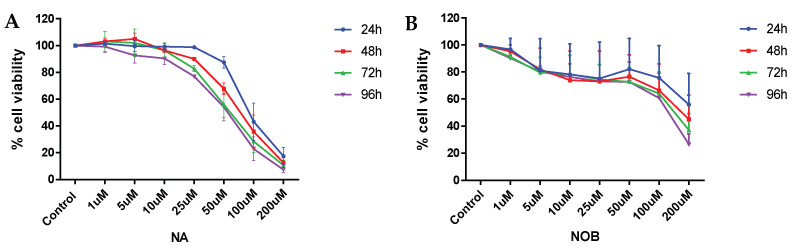
MTT assay was performed on hNPCs following NA and NOB exposure for 24, 48, 72, and 96 h. (**A**) is graphical representation of cell viability with different concentrations of sodium arsenate (NA) and (**B**) showing the cell viability at different concentration of nobiletin (NOB). The values are the mean ± SE of experiment replicates. The data are represented as the mean of the percent of the unexposed control ± SE, *n* = 2 (biological replicates).

Different concentrations of NA and NOB were administered in order to measure cell viability via MTT assay so as to determine the non-cytotoxic doses for our study. Figure 1A represents the NA-induced dose-dependent cytotoxic effects in hNPCs. The viable cell was 67.7% at 50 μM conc. (*p* < 0.01) of NA after 48 h of exposure and 55.8% at 50 μM conc. (*p* < 0.0001) of NA after 72 h of exposure; therefore, the 50 μM concentration for 48 h was adopted as the non-cytotoxic concentration for further experiments. A similar experiment was carried out for NOB. As depicted in Figure 1B, NOB at ≤50 μM did not show any significant toxicity, even in hNPCs exposed for up to 96 h (i.e., >70% viability was maintained), in contrast to the control group. Hence, we selected a concentration of 50 μM of nobiletin to study its neuroprotective impact against NA-mediated cellular toxicity. We therefore selected doses of NA 50 μM, NOB 50 μM, and co-exposure of NA 50 μM + NOB 50 μM for 48 h to further investigate the protective effect of NOB against NA-induced toxic insult.

### 2.2. Nobiletin (NOB) Protects hNPCs against Sodium Arsenate (NA)-Induced Neurotoxicity

**Figure 2:**
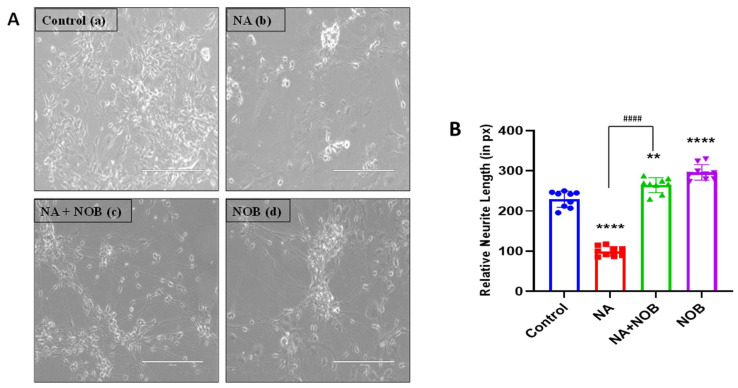
Neuronal protection in hNPCs: (**A**). (**a**) Morphological control, (**b**) Morphological alterations following the exposure to sodium arsenate (NA), (**c**) Morphological alterations/recovery/protection following the co-exposure to NA + NOB, and (**d**) morphological alterations following the exposure to NOB. All images are of phase contrast microscopy, which represents the biological triplicates. (**B**). Graphical representation of neurite length analysis following the different groups of exposures. Data are presented as the ± SEM, *n* = 3 (biological replicates including their technical replicates); the significant changes in comparison to the unexposed control group are: ** *p* < 0.01, **** *p* < 0.0001, and ^####^ *p* < 0.0001 compared with NA-exposed cells. Scale bar, 200 µM. The images were taken at a magnification of 200×.

After selecting non-cytotoxic concentrations for NA and NOB from the cell viability assay, we exposed the cells to NA, NOB, and NA + NOB (co-exposure) (Figure 2A). We observed the neurite length of the hNPCs for every group. The neurite length of the control hNPCs was found to be 229.4 ± 6.7. The neurite length was increased in cells exposed to only NOB (296.1 ± 6.4) in comparison to the control. The cells exposed to NA showed deformations and a decrease in neurite length (99.8 ± 4.0). The cells exposed to NA + NOB (co-exposure) showed increases in neurite length (264.5 ± 6.2) compared to those exposed to NA only. The morphological results indicate a possible neuroprotective effect of nobiletin against NA insult. Exposure to NA significantly reduced neurite length (2.3 times lower than in the control) in hNPCs, but co-exposure to NOB significantly restored neurite length (2.6 times greater than NA-exposed cells), as shown in Figure 2B.

### 2.3. Antioxidative Properties of Nobiletin 

**Figure 3:**
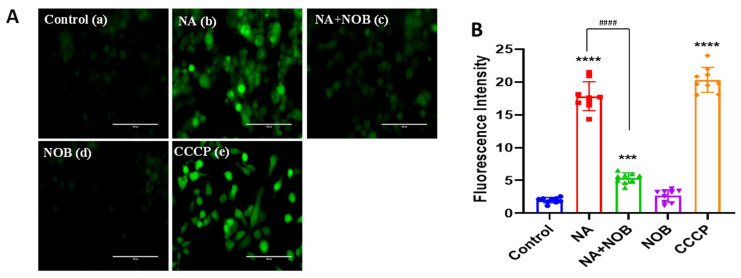
Detection of ROS generation by DCFH-DA in hNPCs (**A**) (**a**) Control or unexposed cells, (**b**) NA-exposed cells, (**c**) NA and NOB co-exposed group, (**d**) NOB exposed cells, and (**e**) Positive control group exposed to CCCP. Images were taken with Invitrogen EVOS FL fluorescence microscope. Scale bar, 100 µM. (**B**) Graphical representation of ROS generation following the different groups of exposures. Cellular and mitochondrial ROS generation was calculated by ImageJ software. Data are represented as the ±SEM of three separate experiment replicates performed in *n* = 3 (biological replicates); the significant changes in comparison to the unexposed control group are *** *p* < 0.001, **** *p* < 0.0001, and ^####^ *p*< 0.0001 compared with NA-exposed cells.

For dissecting out the anti-oxidative activity of NOB in hNPCs, we performed a DCFDA/H_2_DCFDA assay to measure ROS generation. The nonfluorescent H_2_DCFDA transforms to the highly fluorescent 2’,7’-dichlorofluorescein (DCF) after the cleavage of the acetate groups by intracellular esterases and oxidation. The fluorescent intensity of DCF directly correlates to ROS levels. Specifically, increase in the fluorescent intensity of DCF implies the ROS generation in cells. In this experiment, hNPCs were exposed to NA, NOB, and NA + NOB (co-exposure) for 48 h, followed by DCFDA incubation (Figure 3A). Cells exposed to NOB alone showed non-significant ROS generation (2.628 ± 0.31) in comparison to the control (1.972 ± 0.14), while in NA-exposed cells, the fluorescent intensity of DCF was found to be elevated (17.82 ± 0.73); the level was around eight times higher than that of unexposed cells, and this result showed the significant increase in ROS generation in the NA group. The cells exposed to NA + NOB (co-exposure) showed the levels of DCFDA fluorescent intensity (5.34 ± 0.26) that was around three times lower in comparison to NA-exposed cells. The results showed that NA-exposed hNPCs generated the highest ROS levels, while hNPCs co-exposed to NA + NOB showed a significant decrease in ROS generation, indicating the protective efficacy of NOB against NA-induced ROS generation. Carbonyl cyanide m-chlorophenylhydrazone (CCCP) was taken as a positive control that showed maximum ROS generation, i.e., (20.32 ± 0.63) (Figure 3B).

### 2.4. Protective effect of NOB against NA-induced MMP alteration

**Figure 4:**
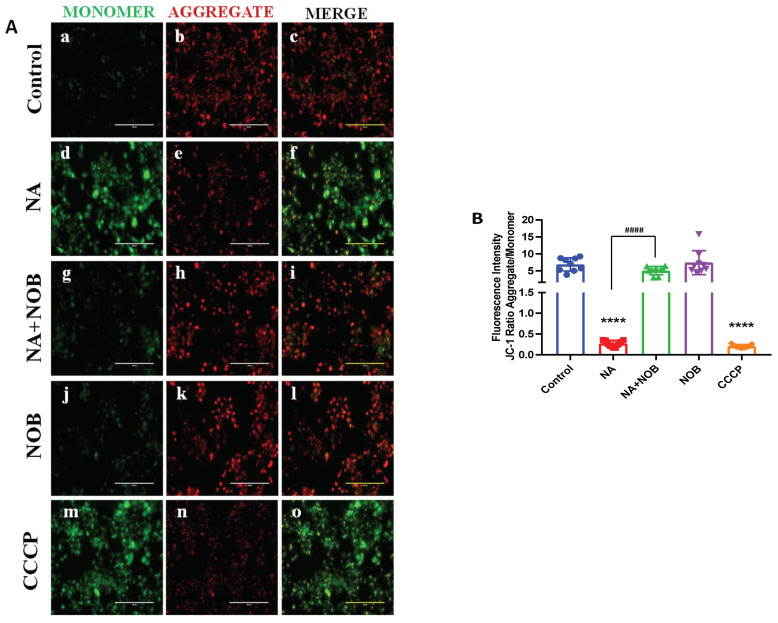
Mitochondrial membrane potential (MMP) measurement by JC1 dye in (**A**). (**c**) Control or unexposed cells, (**f**) NA-exposed cells, (**i**) NA + NOB co-exposed group, (**l**) NOB exposed cells, and (**o**) Positive control group exposed to CCCP. The JC1 aggregates were observed to be red and monomers to be green. Images were taken with an Invitrogen EVOS FL fluorescence microscope. Scale bar, 100 µM. (**B**) Fluorescent intensity was calculated by ImageJ software; images are represented in graph format. Data are presented as the ±SEM of three separate experiments performed in *n* = 3 (biological replicates). The change in expression pattern is statistically significant as indicated by **** *p* < 0.0001 in comparison to the control and ^####^ *p*< 0.0001 compared with NA-exposed cells.

To investigate the protective effect of NOB on NA-mediated alteration in the MMP, a JC-1 assay was performed. JC-1 gets excited at ≈490 nm and emits wavelength at ≈ 530 ± 40 nm (for JC-1 monomers—green) and at ≈ 580 ± 30 nm (for JC-1 aggregates—red). The membrane depolarization is greater if the monomer to aggregate ratio (530 ± 40: 580 ± 30) is larger. We exposed hNPC cells to NA, NOB, and NA + NOB (co-exposure) for 48 h and further administered incubation with JC-1 dye in all the groups. At a higher mitochondria membrane potential, JC-1 forms aggregates (red), but at low membrane potential, JC-1 forms monomers (green). When mitochondria are physiologically normal, their membrane potential is large, and JC-1 enters rapidly, thus forming aggregates; nevertheless, toxic insult can alter the normal membrane potential, leading to mitochondrial dysfunction and a decrease in membrane potential. The hNPCs in the unexposed group were viable and located in the aggregate (red) zone (red: green 6.85 ± 0.65, Figure 4A(c)). For a positive control, we exposed the hNPCs to CCCP (50 μM), a well-known MMP disrupter, and CCCP increased the cells in the JC-1 monomer area and decreased the size of the JC-1 aggregate area (red: green 0.206 ± 0.01, Figure 4A(o)). An enhancement intensity of JC-1 monomeric cells was observed due to the MMP loss induced by NA (50 μM) treatment (red: green 0.270 ± 0.02, Figure 4A(f)), while control cells showed a reduced number of JC-1’s monomeric form in comparison to the NA-exposed group. The exposure of NOB to hNPC cells did not cause a significant change in the MMP (red: green 7.41 ± 1.17, Figure 4A(l)) in comparison to the control group. The cells exposed to NA + NOB (co-exposure) (5.04 ± 0.40, Figure 4A(i)) showed a decrease in the JC-1 monomeric form in comparison to the NA-exposed cells. Thus, the results demonstrated that NOB protects against the MMP disruption caused by NA in hNPCs.

### 2.5. Impact of NOB on Neuronal Markers and Stress Granule Markers in Neural Progenitor Cells Exposed to NA

**Figure 5:**
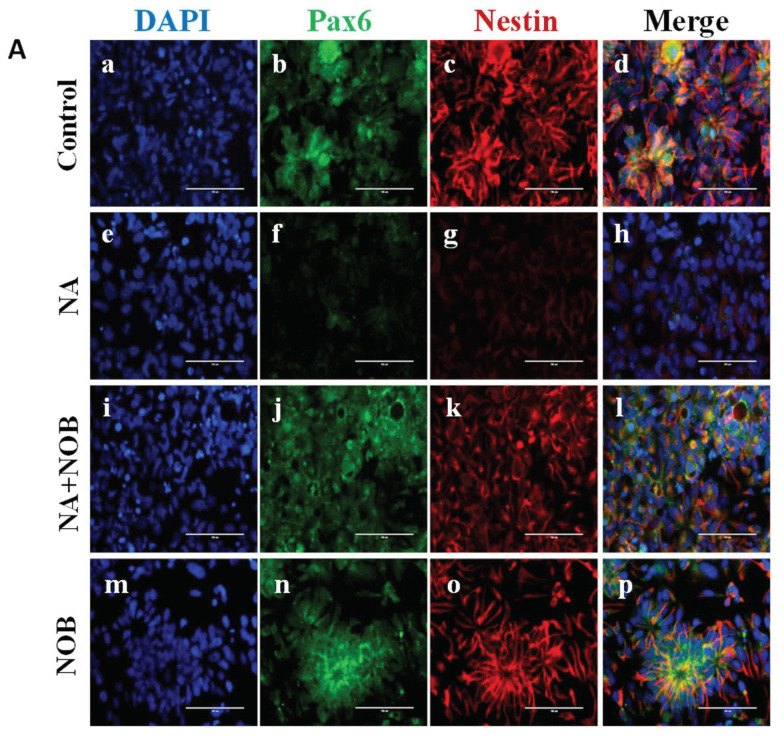

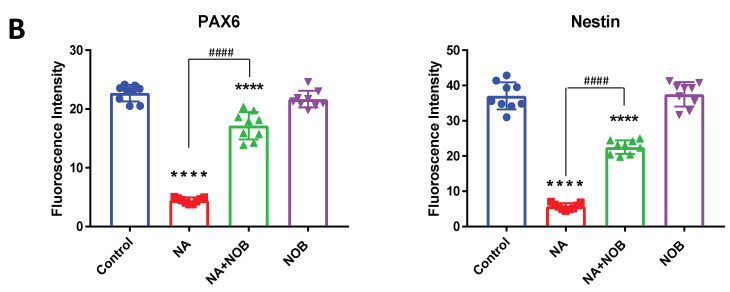
Effect of NA and NOB on hNPCs neuronal markers. (**A**) Representative immunocytochemistry (ICC) images of hNPCs depicting the expression of functional neuronal marker genes PAX6 and nestin of (**d**) Control or unexposed cells, (**h**) NA-exposed cells, (**l**) NA and NOB co-exposed group, (**p**) NOB-exposed cells; Images were taken using an Invitrogen EVOS FL fluorescence microscope. Scale bar, 100 µM. (**B**) Fluorescent intensity was calculated by ImageJ software, and images are represented in graph format. Data are presented as the ±SEM of three separate experiments performed in *n* = 3 (biological replicates). The change in expression pattern is statistically significant as indicated by **** *p* < 0.0001 vs. the control and ^####^ *p* < 0.0001 compared with NA-exposed cells.

We studied the alterations in neuronal markers and stress granule markers via immunocytochemistry techniques where hNPCs were exposed to NA, NOB, and NA + NOB (co-exposure). The procedure followed was performed in accordance with the protocol mentioned in the Materials and Methods section. In Figure 5A, the expression of PAX6 and nestin was shown in all the groups. The fluorescent intensity of the images was calculated and represented in Figure 5B. The cells in the control group that were analyzed for PAX6 had a fluorescence intensity of 22.71 ± 0.47, while the fluorescent intensity in NA-exposed cells was 4.45 ± 0.16, which indicated the presence of neuronal damage in NA-exposed cells in comparison to the control cells. Cells exposed to NOB alone showed a fluorescence intensity of 21.68 ± 0.47, which was relatively similar to the control hNPCs. A significant upregulation of PAX6 fluorescence intensity in co-exposed hNPCs (17.16 ± 0.77) was observed, in contrast with the NA-exposed cells, which confirmed that the identity of the neuronal markers was maintained by NOB. For nestin, NA-exposed cells showed a lower fluorescence intensity of 5.73 ± 0.30 in comparison to the control value of 37.06 ± 1.29, which indicated the impairment of nestin expression in NA-exposed cells. On the other hand, NOB-exposed cells showed a fluorescent intensity of 37.52 ±1.15, which was relatively similar to that of the control group. The NA + NOB co-exposed cells had a fluorescence intensity of 22.54 ± 0.64, which was significantly higher than the NA-exposed group; thus, confirming the protective efficacy of NOB for the restoration of neuronal identity in the case of neuronal damage induced by NA. 

**Figure 6:**
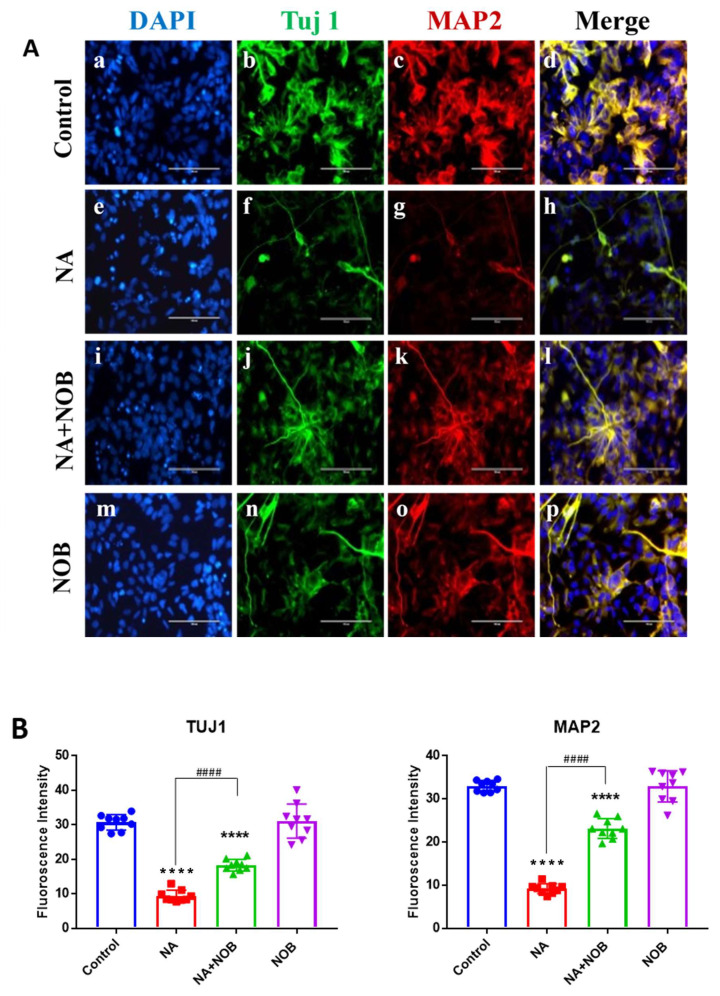
Effect of NA and NOB on hNPCs neuronal markers. Figure (**A**) Representative immunocytochemistry (ICC) images of hNPCs, depicting the expression of the functional neuronal marker genes TUJ1 and MAP2 of (**d**) Control or unexposed cells, (**h**) NA-exposed cells, (**l**) NA and NOB co-exposed group, and (**p**) NOB exposed cells; Images were taken using an Invitrogen EVOS FL fluorescence microscope. Scale bar, 100 µM. (**B**) Fluorescent intensity was calculated by ImageJ software, and images are represented in graph format. Data are presented as the ±SEM of three separate experiments performed in *n* = 3 (biological replicates). The change in expression pattern is statistically significant as indicated by * *p* < 0.05, **** *p* < 0.0001 vs. control and ^####^ *p*< 0.0001 compared with NA-exposed cells.

We also confirmed the expression of Tuj1 and MAP2 via immunocytochemistry techniques (Figure 6). The morphological fluorescence images are represented in Figure 6A, while the calculations of fluorescence intensity are represented in Figure 6B. When cells were exposed to NA, the expression of Tuj1 was found to be 9.37 ± 0.56, while in control cells, it was found to be 30.71 ± 0.73. Results showed neuronal damage in NA-exposed hNPCs and lower levels of neuronal markers expression in comparison to the control. Cells exposed to NOB alone showed a 31.06 ± 1.65 fluorescence intensity, which was very near to that of the control group. The NA + NOB (co-exposure) cells showed an increased fluorescence intensity of 18.3 ± 0.56 in comparison to the NA-exposed cells. This demonstrated that NOB prevented the neuronal marker impairment caused by NA exposure. The MAP2 marker was also analyzed for MAP2 expression in exposed and unexposed cells; the results confirmed the neuroprotective effect of NOB against NA insult. It was shown that cells exposed to NA showed a 9.17 ± 0.38 MAP2 expression in comparison to the control level of 32.99 ± 0.42, whereas in NA + NOB (co-exposure) cells, fluorescence intensity values were found to be 23.15 ± 0.76. The cells exposed to NOB showed a similar value to that of the control group: 32.9 ± 1.20. The results for various neuronal markers showed the restoration efficacy of NOB against NA-mediated neuronal damage in hNPCs.

**Figure 7:**
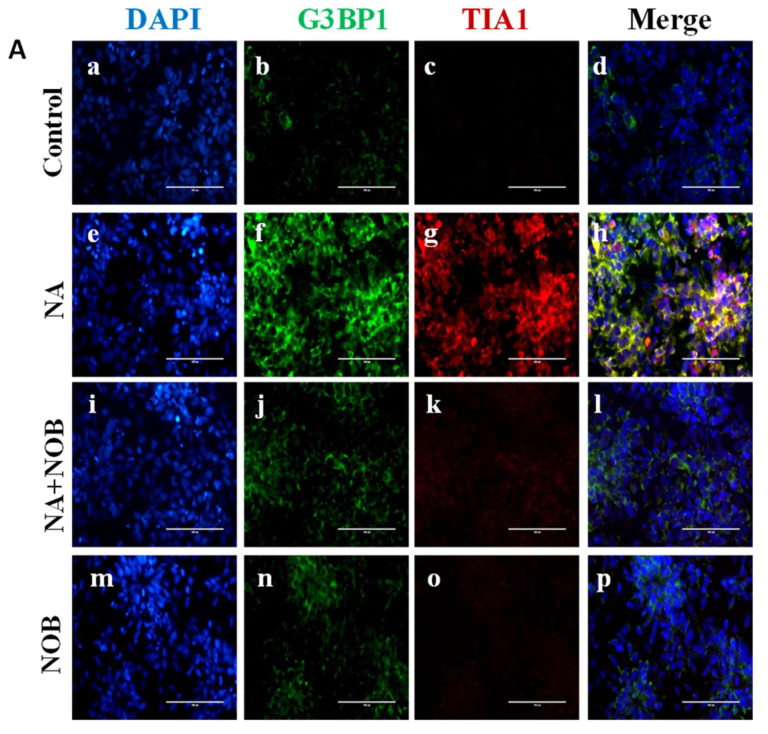

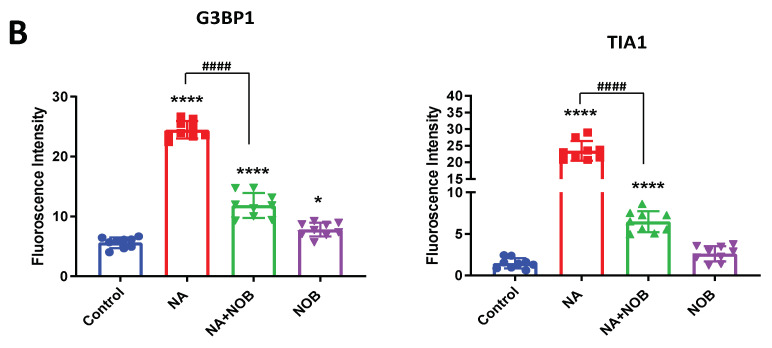
Effect of NOB and NA on stress granule markers G3BP1 and TIA1 in hNPCs. (**A**) Representative immunocytochemistry (ICC) images of hNPCs depicting the expression of G3BP1 and TIA1 positive granules in (**d**) Control or unexposed cells, (**h**) NA-exposed cells, (**l**) NA and NOB co-exposed group, and (**p**) NOB exposed cells; Images were taken using an Invitrogen EVOS FL fluorescence microscope. Scale bar, 100 µM. (**B**). Quantification of cells with G3BP1 and TIA1 positive granules under the indicated conditions. Fluorescent intensity was calculated by ImageJ software, and images are represented in graph format. Data are presented as the ±SEM of three separate experiments performed in 3 biological replicates. The change in expression pattern is statistically significant as indicated by * *p* < 0.05, **** *p* < 0.0001 compared to the control and ^####^ *p* < 0.0001 compared with NA-exposed cells.

The stress granule markers (G3BP1 and TIA1) were analyzed by immunocytochemistry techniques. G3BP1 and TIA1 expressions are presented in Figure 7. The images are represented in Figure 7A, and their calculated fluorescence intensities are shown in Figure 7B. When cells were exposed to NA, the fluorescence intensity was found to be 24.47 ± 0.48 for G3BP1, which was around four times higher than the control value of 5.60 ± 0.29. The results showed the significant enhancement of stress granule marker expression in the presence of toxic insult. NA + NOB (co-exposure) cells showed an 11.85 ± 0.69 fluorescence intensity, which was around two times lower than the cells exposed to NA alone. The resultant decreased intensity showed that NOB along with NA reduced the expression of stress granule markers. The cells exposed to NOB demonstrated a non-significant expression in terms of fluorescence intensity, i.e., 7.79 ± 0.38, in comparison to the control. The expression of the TIA1 stress granule marker was also evaluated, and the results showed that NA-exposed cells had a 23.46 ± 0.98 fluorescence intensity, which was much higher in comparison to the control cells that had a 1.45 ± 0.20 fluorescence intensity. The expression of the stress granule marker TIA1 in NA-exposed cells was found to be approximately 12 times higher than in control exposed cells. NA+ NOB (co-exposed) cells exhibited a fluorescence intensity of 6.46 ± 0.42. This value was around three times lower than NA-exposed cells. This result confirmed that NOB drove the protective effects against the stress granule markers in hNPCs. Thus, it can be stated that NOB has neuroprotective efficacy and an anti-stress potential against toxic insults of NA.

## 3. Discussion

In neuropathological situations, the production of endogenous oxidative stress and reactive oxygen species (ROS) in the central nervous system (CNS) are leading causes of neurodegeneration [34]. Imbalances in oxidative and anti-oxidative responses, along with ROS formation and mitochondrial malfunction, are hypothesized to be involved in the pathophysiology of neuronal cell death [35]. According to our findings, the oxidative state plays a vital role in regulating and controlling neuronal cell survival in the brain by interacting with cellular components. Additionally, the role of mitochondrial dysfunction has also been linked to cellular damage, as depicted in Figure 4, where results showed that NOB protects against the MMP disruption caused by NA in hNPCs. In our investigation, it was also found that sodium arsenate impaired neuronal markers, such Tuj-1 and nestin, but these markers were restored when hNPCs were co-exposed to NA and NOB. The toxicity induced by NA has previously been linked to the production of oxidative stress, which results in neuronal damage and elevated levels of stress granule markers. In our present study, we investigated the role of TIA1 and G3BP1 stress granule markers in NA-induced neurotoxicity. TIA1 aids in the recruitment of the spliceosome, which regulates RNA splicing, and suppresses the transcription of several genes, including the cytokine tumor necrosis factor-alpha. TIA1 moves from the nucleus to the cytoplasm in response to stress, where it nucleates a form of RNA granule known as the stress granule; hence, TIA1 participates in the translational stress response [36]. G3BP1 is a multifunctional protein best known for its role in stress granule construction and related dynamics. Evidence suggests that G3BP1 appears necessary for neural growth and survival [37]. The creation of G3BP1/mice exhibiting late embryonic lethality emphasizes the relevance of G3BP1. All organs were present and were of standard size in G3BP1/neonates (on the 129/Sv mouse background), with the only notable anomaly being significant cell death in the brain. Many follow-up studies focusing on stress granule dynamics have been carried out in immortalized cell lines (e.g., HeLa, U2OS, HEK293) or using over-expression to express proteins prone to phase separation [38]. In this study, perhaps for the first time, we investigated the involvement of G3BP1- and TIA1-linked neuronal impairment in in vitro hNPCs exposed to NA to determine the impact of oxidative stress and evaluate the anti-oxidative effect of NOB. It was discovered that when cells were exposed to NA, the stress granule markers TIA1 and G3BP1 were upregulated, and when cells were co-exposed to NA and NOB, it ameliorated the level of stress granule markers; hence, it can be inferred that NOB aided in neuronal restoration and provided protection from oxidative stress.

According to a prior study, nobiletin improved memory in a rat AD model by repairing the phosphorylation of the cAMP response element-binding protein, which had been damaged due to amyloid formation. The Alzheimer’s Disease Assessment Scale-Cognitive Subscale (ADAS-J cog) was used in a case study to see whether nobiletin could delay the progression of cognitive impairment in donepezil-administered AD patients [39]. The blood–brain barrier (BBB) permeability of NOB was examined in several studies that reported that it has high BBB permeability, and the maximal concentrations of nobiletin in the brain and plasma were found to be 4.20 µg/mL and 1.78 µg/mL, respectively [40,41]. Hence, nobiletin could be considered as a possible promising compound for managing brain cell impairment. A study by Lan Zhang. et al. stated that nobiletin stimulates antioxidant and anti-inflammatory responses and protects against ischemic stroke. The authors demonstrated that the ischemia-induced upregulation of NF-κB and MMP9 were reduced following NOB injection, suggesting that NOB’s therapeutic impact in ischemic stroke may be linked to a lower expression of MMP9 and NF-κB genes [24]. Filiz Kazak. et al. discovered that nobiletin treatment boosted BDNF levels and prevented cisplatin-induced neuronal degeneration and edema. In a rat model of cisplatin-induced neurotoxicity, it also reduced cisplatin-induced apoptosis in the cerebrovascular endothelium and neurons. Thus, nobiletin might be considered as a supplement to cisplatin therapy for cancer patients [42]. Our results showed a substantial reduction in the reactive oxygen species and an amelioration of the mitochondrial membrane potential in the co-exposure group (NA + NOB) in comparison to the NA-exposed group. Hence, it can be concluded that NOB could be a potential agent for managing and protecting neuronal cells from oxidative damage caused by NA exposure.

**Figure 8:**
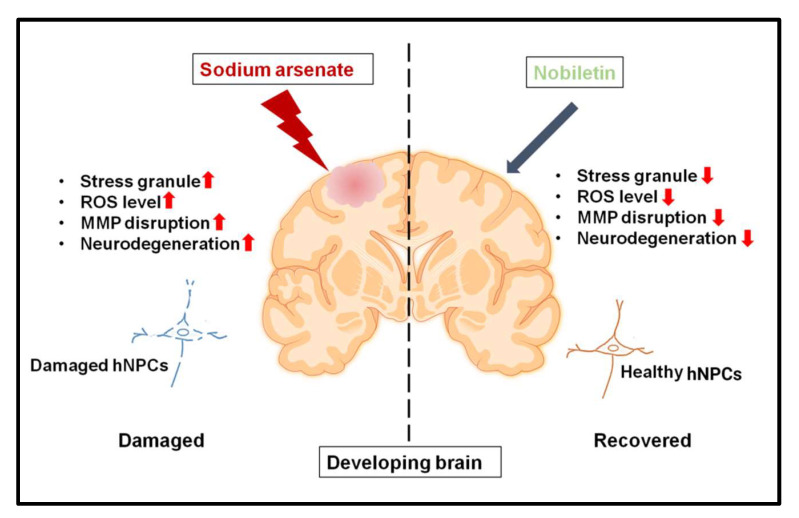
Schematic representation of NA-induced neuronal damage and the neuroprotective efficacy of NOB on human neural progenitor cells (hNPCs). (Figure Created with BioRender.com (accessed on 19 April 2022).

Pax6 regulates the fate of neural progenitor cells throughout their development and guides them to adult neuronal commitment [43]. Pax6 is required in mammals to determine the neuro-ectodermal fate from pluripotent embryonic cells, for neurogenesis, and also for maintaining the stem cell and progenitor cell population in the actively dividing germinal core [44]. Pax6 expression begins in a broad region of the neuro-epithelium in mice as early as embryonic day 8, when the neural tube approaches closure. Pax6 is expressed throughout neuronal development, from the actively dividing germinal core across the cellular regions of the embryonic forebrain, midbrain, hindbrain, and adult brain [45,46]. The aforementioned Pax6 functions are based on its expression patterns in different brain areas throughout neuronal development. In this study, we looked at PAX-6 protein expression in hNPCs and discovered that NA downregulates PAX-6 expression, whereas NOB increased PAX-6 expression, indicating the neuroprotective role of nobiletin. The microtubule-associated protein 2 (MAP2) family of proteins, in particular, is a large family of cytoskeletal components that are primarily produced in neurons and act as substrates for the majority of protein kinases and phosphatases [47]. MAP2 plays a crucial role in the nucleation and stabilization of microtubules, the regulation of organelle translocation within axons and dendrites, and the anchoring of regulatory proteins such as protein kinases, all of which is significant for signal transmission. The function of MAP2 is related to the expansion of neuronal processes, synaptic plasticity, and neuronal cell death. As a result, MAP2 is a fascinating case study for understanding how its expression regulates neuronal function [48,49]. To corroborate the presence of MAP-2, its expression was examined in control and exposed cells using immunocytochemistry. We discovered that MAP-2 was downregulated in NA-exposed cells, whereas hNPCs co-exposed to NA + NOB showed higher expression of MAP2, confirming the neuroprotective efficacy of NOB. Thus, NOB may play a crucial role in the maintenance of neural integrity when altered by increased oxidative stress in response to NA exposure. Moreover, our findings also indicated that the neuroprotective action of NOB prevented the loss of other neuronal markers, such as nestin and Tuj1, when exposed to NA. Therefore, NOB can be marked as a potential neuroprotectant with anti-oxidative and anti-stress properties for the management and cure of neurodegenerative pathologies. A schematic representation depicting the neuroprotective role of NOB against NA-induced neuronal damage is illustrated in Figure 8. However, there is much scope for further elucidating the molecular mechanisms of the action of nobiletin with respect to the protection of neuronal cells from xenobiotic-induced oxidative stress and neuronal damage.

## 4. Materials and Methods

### 4.1. Reagents and Consumables

Chemicals reagents and kits used were procured from Thermo Fisher Scientific (Waltham, MA, USA) and sigma unless and otherwise stated. Essential 8 medium (#A1517001), DMEM/F12 medium (#12500062), N2 supplement (#17502048), B27 supplement (#17504044), Invivogen Normocin (#ant-nr-1), Episomal human iPSCs (#A18945), sodium biocarbonate, fluorescent antibodies, and D-PBS were purchased from Gibco (Thermo Fisher Scientific). All the primary antibodies were purchased from abcam. Sodium arsenate and carbonyl cyanide m-chlorophenylhydrazone (CCCP) were purchased from sigma-aldrich. Nobiletin was procured from carbosynth. Plastic and culture wares were procured from Thermofisher scientific and Corning Inc. All the experiments were performed using Autoclaved Milli Q water.

### 4.2. Cell Culture

Human episomal iPSC cell lines were obtained from Gibco. A standard cell culture medium, Essential 8™ Medium, was used for culturing hiPSCs. A matrigel-coated 60 mm culture dish was used that provided a defined surface for a feeder-free extracellular matrix, thus maintaining pluripotency and average growth of iPSCs. The iPSCs were cultured on matrigel-coated 60 mm dish in Essential 8 medium, and kept in a humidified incubator with 5% CO_2_ at 37 °C. For the formation of neural progenitor cells (hNPCs), pure iPSC colonies were detached manually and grown in suspension in an ultra-low attachment plate to form embryoid bodies (EBs). Large spherical EBs were selected and plated over the matrigel-coated culture dish to form hNPCs. hNPCs were cultured in a 60 mm dish and expanded for the experiments.

### 4.3. MTT Assay

The MTT test was carried out by the procedure for assessing NA- and NOB-induced cytotoxicity [50]. Approximately 20,000 hNPC cells were seeded in a matrigel-coated 96-well culture plate and incubated in a humidified incubator with 5% CO_2_ at 37 °C. On the next day, the cells were starved by giving a basal medium of DMEM/F12 only. Cells were subjected to different doses (1 µM, 5 µM, 10 µM, 25 µM, 50 µM, 100 µM, and 200 µM) of NA and NOB. At periods of 24 h, 48 h, 72 h, and 96 h, the cytotoxicity induced by NA and NOB was measured. When it was 4 h before the end of the dose exposure time, 10 µL of MTT (5 mg/mL) was added to each well and incubated for approx. 4 h at 37 °C, 5% CO_2_. The MTT-containing media was aspirated after exposure, and formazan crystals were dissolved using 200 µL DMSO. The plate was read at 550 nm using a spectrophotometer (Synergy HT, BioTek, Santa Clara, USA). The trials were carried out in triplicate, with a positive control set running concurrently.

### 4.4. Morphology of Cells and Neurite Length

After selecting the concentrations of NA and NOB, cells were treated with NA 50 µM, NOB 50 µM, and the co-exposure of NA and NOB for 48 h. A phase-contrast microscope equipped with NIS Element BR software from Nikon (Melville, NY, USA) was used for the measurement of neurite length.

### 4.5. ROS Generation

The 2′,7′-dichlorodihydrofluorescein diacetate (DCF-DA) fluorescent dye was used to detect ROS generation quantitatively and qualitatively. The substrate was 2′,7′ dichlorofluorescein diacetate, which was measured when oxidized into 2′,7′ dichlorofluorescein (DCF). In brief, the control and exposed groups of hNPCs were incubated in the dark at 37 °C for 60 min after adding 10 μM DCF-DA. For the positive control, a 50 μM final concentration of carbonyl cyanide m-chlorophenylhydrazone (CCCP) was given to the cells and incubated for 5 min at 37 °C. The intracellular fluorescence of DCF was measured using the Invitrogen EVOS FL imaging system. The experiments were carried out in triplicate.

### 4.6. JC-1 Dye for Mitochondrial Membrane Potential

The regular mitochondrial activity of cells may be assessed by measuring changes in the mitochondrial membrane potential. In a 6-well culture plate, 1 × 10^5^ cells were plated per well and incubated for 20–30 min at 37 °C, 5% CO_2_. The cells were then exposed to selected doses of NA and NOB. After the completion of 24 h of treatment, the cells were washed in PBS, and 2 μM of JC-1 dye was placed in each well, followed by 15–30 min of incubation at 37 °C with 5% CO_2_. For the positive control, 50 μM final concentration of CCCP was added to the cells and incubated for 5 min at 37 °C. Using Invitrogen EVOS FL imaging, the fluorescence intensity of the test cultures (including controls) was assessed immediately after the dye was replaced with pre-warmed PBS.

### 4.7. Immunocytochemical Analysis

hNPCs were seeded on the matrigel-coated 18 mm glass coverslip on 12 healthy culture plates. Cells were incubated with primary and secondary antibodies, and slides were prepared using Fluoroshield™ with DAPI. The slides were visualized under EVOS FL using specific filters for secondary antibodies and DAPI. Three randomly selected microscopic fields were considered and analyzed for measuring fluorescence intensity using ImageJ (1.49 V) analysis software (NIH Image) for each marker. The AFI values were acquired from the ImageJ software and were manually derived in Microsoft Excel. GraphPad Prism 7.0 software (GraphPad Software, La Jolla, CA, USA) was used for the data analysis.

### 4.8. Statistical Analysis

The experiments were performed in triplicate, and data were represented as the ± standard error of the mean (SEM). Comparisons among the groups were performed using one-way analysis of variance (ANOVA). The GraphPad Prism 7 application was used to interpret the corresponding parameters, error bars, *p*-values, and bar graphs.

## Data Availability

Data is contained within the article.

## References

[1] World Health Organization (2019). Preventing Disease through Healthy Environments: Exposure to Arsenic: A Major Public Health Concern.

[2] Keune K., Mass J., Meirer F., Pottasch C., van Loon A., Hull A., Church J., Pouyet E., Cotte M., Mehta A. (2015). Tracking the transformation and transport of arsenic sulfide pigments in paints: Synchrotron-based X-ray micro-analyses. J. Anal. At. Spectrom..

[3] Mandal B.K., Suzuki K.T. (2002). Arsenic round the world: A review. Talanta.

[4] Ahmad S.A., Khan M.H., Haque M. (2018). Arsenic contamination in groundwater in Bangladesh: Implications and challenges for healthcare policy. Risk Manag. Healthc. Policy.

[5] Schuhmacher–Wolz U., Dieter H.H., Klein D., Schneider K. (2009). Oral exposure to inorganic arsenic: Evaluation of its carcinogenic and non-carcinogenic effects. Crit. Rev. Toxicol..

[6] Powers M., Sanchez T.R., Grau-Perez M., Yeh F., Francesconi K.A., Goessler W., George C.M., Heaney C., Best L.G., Umans J.G. (2019). Low-moderate arsenic exposure and respiratory in American Indian communities in the Strong Heart Study. Environ. Health.

[7] Rodrigues E.G., Bellinger D.C., Valeri L., Hasan M.O.S.I., Quamruzzaman Q., Golam M., Kile M.L., Christiani D.C., Wright R.O., Mazumdar M. (2016). Neurodevelopmental outcomes among 2-to 3-year-old children in Bangladesh with elevated blood lead and exposure to arsenic and manganese in drinking water. Environ. Health.

[8] Gong G., O’Bryant S.E. (2010). The arsenic exposure hypothesis for Alzheimer disease. Alzheimer Dis. Assoc. Disord..

[9] Tsuji J.S., Garry M.R., Perez V., Chang E.T. (2015). Low-level arsenic exposure and developmental neurotoxicity in children: A systematic review and risk assessment. Toxicology.

[10] Sarkar S., Mukherjee S., Chattopadhyay A., Bhattacharya S. (2014). Low dose of arsenic trioxide triggers oxidative stress in zebrafish brain: Expression of antioxidant genes. Ecotoxicol. Environ. Saf..

[11] García-Chávez E., Segura B., Merchant H., Jiménez I., Del Razo L.M. (2007). Functional and morphological effects of repeated sodium arsenite exposure on rat peripheral sensory nerves. J. Neurol. Sci..

[12] Garza-Lombó C., Posadas Y., Quintanar L., Gonsebatt M.E., Franco R. (2018). Neurotoxicity linked to dysfunctional metal ion homeostasis and xenobiotic metal exposure: Redox signaling and oxidative stress. Antioxidants Redox Signal..

[13] Srivastava P., Dhuriya Y.K., Kumar V., Srivastava A., Gupta R., Shukla R.K., Yadav R.S., Dwivedi H.N., Pant A.B., Khanna V.K. (2018). PI3K/Akt/GSK3β induced CREB activation ameliorates arsenic mediated alterations in NMDA receptors and associated signaling in rat hippocampus: Neuroprotective role of curcumin. Neurotoxicology.

[14] Sharma A., Kshetrimayum C., Sadhu H.G., Kumar S. (2018). Arsenic-induced oxidative stress, cholinesterase activity in the brain of Swiss albino mice, and its amelioration by antioxidants Vitamin E and Coenzyme Q10. Environ. Sci. Pollut. Res..

[15] Salim S. (2017). Oxidative stress and the central nervous system. J. Pharmacol. Exp. Ther..

[16] Shila S., Kokilavani V., Subathra M., Panneerselvam C. (2005). Brain regional responses in antioxidant system to α-lipoic acid in arsenic intoxicated rat. Toxicology.

[17] Mateju D., Franzmann T.M., Patel A., Kopach A., Boczek E.E., Maharana S., Lee H.O., Carra S., Hyman A.A., Alberti S. (2017). An aberrant phase transition of stress granules triggered by misfolded protein and prevented by chaperone function. EMBO J..

[18] Protter D.S., Parker R. (2016). Principles and properties of stress granules. Trends Cell Biol..

[19] Wei S.C., Fattet L., Tsai J.H., Guo Y., Pai V.H., Majeski H.E., Chen A.C., Sah R.L., Taylor S.S., Engler A. (2015). Matrix stiffness drives epithelial–mesenchymal transition and tumour metastasis through a TWIST1–G3BP2 mechanotransduction pathway. Nat. Cell Biol..

[20] Panas M.D., Ivanov P., Anderson P. (2016). Mechanistic insights into mammalian stress granule dynamics. J. Cell Biol..

[21] Omidifar N., Nili-Ahmadabadi A., Nakhostin-Ansari A., Lankarani K.B., Moghadami M., Mousavi S.M., Hashemi S.A., Gholami A., Shokripour M., Ebrahimi Z. (2021). The modulatory potential of herbal antioxidants against oxidative stress and heavy metal pollution: Plants against environmental oxidative stress. Environ. Sci. Pollut. Res..

[22] Youn K., Lee S., Jun M. (2019). Discovery of nobiletin from citrus peel as a potent inhibitor of β-amyloid peptide toxicity. Nutrients.

[23] Wang M., Meng D., Zhang P., Wang X., Du G., Brennan C., Li S., Ho C.-T., Zhao H. (2018). Antioxidant protection of nobiletin, 5-demethylnobiletin, tangeretin, and 5-demethyltangeretin from citrus peel in Saccharomyces cerevisiae. J. Agric. Food Chem..

[24] Zhang L., Zhang X., Zhang C., Bai X., Zhang J., Zhao X., Chen L., Wang L., Zhu C., Cui L. (2016). Nobiletin promotes antioxidant and anti-inflammatory responses and elicits protection against ischemic stroke in vivo. Brain Res..

[25] Nakajima A., Yamakuni T., Haraguchi M., Omae N., Song S.-Y., Kato C., Nakagawasai O., Tadano T., Yokosuka A., Mimaki Y. (2007). Nobiletin, a citrus flavonoid that improves memory impairment, rescues bulbectomy-induced cholinergic neurodegeneration in mice. J. Pharmacol. Sci..

[26] Nakajima A., Aoyama Y., Shin E.-J., Nam Y., Kim H.-C., Nagai T., Yokosuka A., Mimaki Y., Yokoi T., Ohizumi Y. (2015). Nobiletin, a citrus flavonoid, improves cognitive impairment and reduces soluble Aβ levels in a triple transgenic mouse model of Alzheimer’s disease (3XTg-AD). Behav. Brain Res..

[27] Onozuka H., Nakajima A., Matsuzaki K., Shin R.-W., Ogino K., Saigusa D., Tetsu N., Yokosuka A., Sashida Y., Mimaki Y. (2008). Nobiletin, a citrus flavonoid, improves memory impairment and Aβ pathology in a transgenic mouse model of Alzheimer’s disease. J. Pharmacol. Exp. Ther..

[28] Nakajima A., Aoyama Y., Nguyen T.-T.L., Shin E.-J., Kim H.-C., Yamada S., Nakai T., Nagai T., Yokosuka A., Mimaki Y. (2013). Nobiletin, a citrus flavonoid, ameliorates cognitive impairment, oxidative burden, and hyperphosphorylation of tau in senescence-accelerated mouse. Behav. Brain Res..

[29] Lu Y.-H., Su M.-Y., Huang H.-Y., Yuan C.-G. (2010). Protective effects of the citrus flavanones to PC12 cells against cytotoxicity induced by hydrogen peroxide. Neurosci. Lett..

[30] Cho H.W., Jung S.Y., Lee G.H., Cho J.H., Choi J.H. (2015). Neuroprotective effect of Citrus unshiu immature peel and nobiletin inhibiting hydrogen peroxide-induced oxidative stress in HT22 murine hippocampal neuronal cells. Pharmacogn. Mag..

[31] Masjosthusmann S., Siebert C., Hübenthal U., Bendt F., Baumann J., Fritsche E. (2019). Arsenite interrupts neurodevelopmental processes of human and rat neural progenitor cells: The role of reactive oxygen species and species-specific antioxidative defense. Chemosphere.

[32] Tyler C.R., Allan A.M. (2013). Adult hippocampal neurogenesis and mRNA expression are altered by perinatal arsenic exposure in mice and restored by brief exposure to enrichment. PLoS ONE.

[33] Marchetto M.C., Carromeu C., Acab A., Yu D., Yeo G.W., Mu Y., Chen G., Gage F.H., Muotri A.R. (2010). A model for neural development and treatment of Rett syndrome using human induced pluripotent stem cells. Cell.

[34] He J., Zhu G., Wang G., Zhang F. (2020). Oxidative stress and neuroinflammation potentiate each other to promote progression of dopamine neurodegeneration. Oxidative Med. Cell. Longev..

[35] Khatoon R., Pahuja M., Parvez S. (2020). Cross talk between mitochondria and other targets in Alzheimer’s disease. J. Environ. Pathol. Toxicol. Oncol..

[36] Lourou N., Gavriilidis M., Kontoyiannis D. (2019). Lessons from studying the AU-rich elements in chronic inflammation and autoimmunity. J. Autoimmun..

[37] Sidibé H., Dubinski A., Vande Velde C. (2021). The multi-functional RNA-binding protein G3BP1 and its potential implication in neurodegenerative disease. J. Neurochem..

[38] Sidibé H., Vande Velde C. (2019). RNA granules and their role in neurodegenerative diseases. The Biology of mRNA: Structure and Function.

[39] De Felice F.G., Wasilewska-Sampaio A.P., Barbosa A.C.A., Gomes F.C., Ferreira S.T. (2007). Cyclic AMP enhancers and Aβ oligomerization blockers as potential therapeutic agents in Alzheimer’s disease. Curr. Alzheimer Res..

[40] Braidy N., Behzad S., Habtemariam S., Ahmed T., Daglia M., Mohammad Nabavi S., Sobarzo-Sanchez E., Fazel Nabavi S. (2017). Neuroprotective effects of citrus fruit-derived flavonoids, nobiletin and tangeretin in Alzheimer’s and Parkinson’s disease. CNS Neurol. Disord.-Drug Targets.

[41] Shimazu R., Anada M., Miyaguchi A., Nomi Y., Matsumoto H. (2021). Evaluation of Blood–Brain Barrier Permeability of Polyphenols, Anthocyanins, and Their Metabolites. J. Agric. Food Chem..

[42] Kazak F., Akalın P.P., Yarım G.F., Başpınar N., Özdemir Ö., Ateş M.B., Altuğ M.E., Deveci M.Z.Y. (2021). Protective effects of nobiletin on cisplatin induced neurotoxicity in rats. Int. J. Neurosci..

[43] Blake J.A., Ziman M. (2014). Pax genes: Regulators of lineage specification and progenitor cell maintenance. Development.

[44] Semrau S., Goldmann J.E., Soumillon M., Mikkelsen T.S., Jaenisch R., Van Oudenaarden A. (2017). Dynamics of lineage commitment revealed by single-cell transcriptomics of differentiating embryonic stem cells. Nat. Commun..

[45] Duan D., Fu Y., Paxinos G., Watson C. (2013). Spatiotemporal expression patterns of Pax6 in the brain of embryonic, newborn, and adult mice. Anat. Embryol..

[46] López J.M., Morona R., Moreno N., Lozano D., Jimenez S., González A. (2020). Pax6 expression highlights regional organization in the adult brain of lungfishes, the closest living relatives of land vertebrates. J. Comp. Neurol..

[47] Avila J., Lucas J.J., Perez M., Hernandez F. (2004). Role of tau protein in both physiological and pathological conditions. Physiol. Rev..

[48] Witte H., Bradke F. (2008). The role of the cytoskeleton during neuronal polarization. Curr. Opin. Neurobiol..

[49] Grimes C.A., Jope R.S. (2001). The multifaceted roles of glycogen synthase kinase 3β in cellular signaling. Prog. Neurobiol..

[50] Plumb J.A. (2004). Cell sensitivity assays: The MTT assay. Cancer Cell Culture.

